# Opportunistic Features of Non-*Clostridium botulinum* Strains Containing *bont* Gene Cluster

**DOI:** 10.3390/pathogens13090780

**Published:** 2024-09-10

**Authors:** Tomasz Grenda, Anna Grenda, Anna Jakubczyk, Kamila Rybczyńska-Tkaczyk

**Affiliations:** 1 Department of Hygiene of Animal Feeding Stuffs, National Veterinary Research Institute in Pulawy, Patyzantow 57, 24-100 Pulawy, Poland; 2 Department of Pneumonology, Oncology and Allergology, Medical University in Lublin, ul. Jaczewskiego 8, 20-090 Lublin, Poland; anna.grenda@umlub.pl; 3 Department of Biochemistry and Food Chemistry, University of Life Sciences in Lublin, Skromna 8, 20-704 Lublin, Poland; anna.jakubczyk@up.lublin.pl; 4 Department of Environmental Microbiology, University of Life Sciences in Lublin, St. Leszczyńskiego 7, 20-069 Lublin, Poland; kamila.rybczynska-tkaczyk@up.lublin.pl

**Keywords:** *Clostridium*, BoNT, *C. botulinum*, *C. sporogenes*, *C. butyricum*, *C. baratii*

## Abstract

The cluster of genes determining the production of botulinum toxins is an attribute of not only the *Clostridium botulinum* species. This cluster is also found in other members of the *Clostridium* genus, such as *C. baratii*, *C. butyricum*, and *C. sporogenes*. The occurrence of a botulinum-like cluster has also been recorded in strains of other genera, i.e., *Enterococcus faecium*, as well as in a Gram-negative species isolated from freshwater sediments; however, the biological activity of *bont*-related genes has not been noted. It can be said that the mentioned species have a dual nature. Another species with a dual nature is *C. butyricum*. This bacterium is a common human and animal gut commensal bacterium and is also frequently found in the environment. Although non-toxigenic strains are currently used as probiotics in Asia, other strains have been implicated in pathological conditions, such as botulism in infants or necrotizing enterocolitis in preterm neonates. Additionally, *C. baratii* strains are rare opportunistic pathogens associated with botulism intoxication. They have been isolated from food and soil and can be carried asymptomatically or cause botulism outbreaks in animals and humans. In addition to the mentioned clostridia, the other microorganisms considered as non-toxigenic have also been suspected of carrying botulinum cluster Gram-negative bacteria, such as *Chryseobacterium piperi* isolated from freshwater sediments; however, the biological activity of *bont*-related genes has not been noted. Additionally, *Enterococcus faecium* strains have been discovered carrying BoNT-related clusters (BoNT/En). Literature data regarding the heterogeneity of BoNT-producing strains indicate the requirement to reclassify *C. botulinum* species and other microorganisms able to produce BoNTs or possess botulinum-like gene clusters. This article aims to show the dual nature of *Clostridium* strains not belonging to the *C. botulinum* species that are sporadically able to carry *bont* clusters, which are usually considered saprophytic and even probiotic, and *bont*-like clusters in microorganisms from other genera. The aim was also to consider the genetic mechanisms of botulinum cluster expression in strains that are considered opportunistic and the microbiological safety aspects associated with their occurrence in the environment.

## 1. Introduction

*Clostridium* is a broad group of obligate anaerobes belonging to the *Firmicutes* bacterial phylum. Most of them have a Gram-positive cell wall structure. This genus includes mainly saprophytes. Some strains of this genus are considered probiotics but also significant human and animal pathogens that cause dangerous diseases, such as botulism, gangrene, and tetanus. They produce spores that are resistant to pasteurization temperatures. This genus inhabits the soil and the digestive tract of animals and humans. *Clostridium* is also found in the microflora of the female reproductive system. The genus *Clostridium* is a highly heterogeneous group of bacteria. Most of them are not associated with any pathogenic processes. Most species are saprophytes or even considered probiotics, even though some strains of this species could possess pathogenic features, such as an ability to produce botulinum toxins [1,2].

Historically, botulinum toxin was considered to be produced by *Clostridium botulinum*. Botulism has been affecting human civilization from the earliest time. However, an accurately described outbreak of foodborne botulism was reported as late as the 18th century [3]. The first isolation of *C. botulinum* was conducted in 1895 by Emile Piere van Ermengem. This pathogen was isolated from a salted ham associated with a botulism outbreak. Firstly, this microorganism was named Bacillus Botulinus and was changed further into *C. botulinum* [3]. The chronology of discovered toxinotypes of this bacterium (besides B and A) is indicated by subsequent letters of the alphabet from A to G. The bacterium discovered by van Ermengem was marked as B, and the last as G [4]. Based on these discoveries, a definition was formulated that all clostridia able to produce botulinum toxins are classified to *C. botulinum* species [5]. Due to differences in metabolism and 16S rRNA gene sequences, strains of this pathogen are classified into four metabolic groups, i.e., group I includes all type A and proteolytic type B and F strains, group II includes all type E and non-proteolytic type B and F strains, and group III consists of type C and D and mosaic strains—CD and DC. Group IV includes type G strains. The mentioned groups are related to other species considered to be non-toxigenic, i.e., *C. sporogenes* and *C. tepidum* are deemed related to group I, *C. butyricum*, *C. taeniosporium*, and *C. beijerinckii* to group II, *C. novyi* to group III, and *C. argentinense*, *C. subterminale*, and *C. schirmacherense* to group IV [6,7].

**Figure 1:**
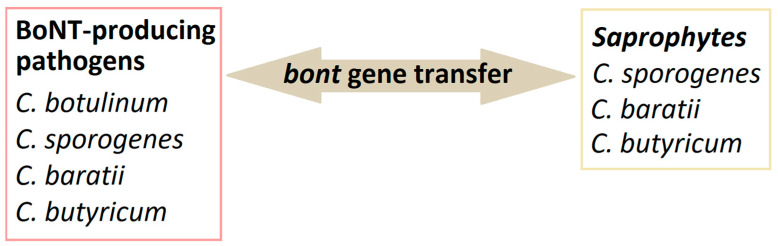
Horizontal *bont* gene transfer among *Clostridium* strains.

Moreover, it has been observed that horizontal transfer of genes determining toxigenicity between strains of *C. botulinum* and related species is possible (Figure 1). The ability to produce botulinum toxins has been previously reported in some strains of *C. butyricum* and *C. baratii* [7]. Some strains of *C. butyricum* can produce BoNT/E. The evidence of toxigenic *C. butyricum* isolation has been noted in the cases of intestinal toxemia botulism in infants and adults in Italy, Japan, the USA, Ireland, and Great Britain [8]. In recent years, it has also been reported in *C. sporogenes* strains [9]. The detection of *C. botulinum* is not possible solely based on biochemical features. It is difficult due to the diversity mentioned above within the genus, i.e., the occurrence of strains phenotypically similar to this species, which cannot produce BoNTs. It should also be noted that the first thing generally determined about a BoNT-producing organism is that it produces a toxin, followed by its toxin type, and then the organism genus and species. Moreover, the toxigenicity of group III strains (toxinotypes C, D and their mosaic CD and DC variants) is determined by the conversion of lysogenic bacteriophages, and the production of toxins by the group I and II strains may be conditioned by horizontal gene transfer [6]. Defining *C. botulinum* as a species is challenging because of the mentioned heterogeneity and phenotypical diversity.

## 2. Saprophytic and Pathogenic *Clostridium sporogenes*

### 2.1. General Clostridium sporogenes Description

*Clostridium sporogenes* is an anaerobic Gram-positive, spore-producing rod found in soil and the human and animal gastrointestinal tract as a part of the normal intestinal flora [10] and is closely related to proteolytic strains of *Clostridium botulinum*. The spoilage of this organism usually results in blown or torn packages with a strong, putrid odor, first described in detail by Metchnikoff in the year 1908. The first strains of *C. sporogenes* have been isolated from the gastrointestinal tract of healthy individuals and those with chronic colitis [11].

*C. sporogenes* species include both pathogenic and saprophytic strains. *C. sporogenes* cause food spoilage due to their genetic and physiological similarity to *Clostridium botulinum* group I. Some strains of both *C. sporogenes* and *C. botulinum* group I produce spores that are highly resistant to heat in the environment. This state of metabolic dormancy combined with heat resistance allows these bacteria to survive in adverse conditions such as lack of nutrients, desiccation, oxygen, high pressure, heat treatment, and toxic chemicals. It is why these bacteria pose severe problems with food spoilage and food safety [12].

### 2.2. Pathogenic Strains of Clostridium sporogenes

Pathogenic activity of *C. sporogenes* strains is most often described in immunocompromised patients, e.g., HIV-positive or cancer patients, COVID patients with pneumonia, or elderly (older than 65 years) people [10,13,14,15,16]. *C. sporogenes* infections have also been detected in patients with leukopenia and renal transplant recipients [12]. The most severe cases associated with pathogenic strains of *C. sporogenes* are related to bacteremia. According to our knowledge, up to now, there have been 29 described infections caused by *C. sporogenes*, including 19 bacteremias, one pyogenic liver abscess, two empyemas, one septic arthritis, two septicaemias [17], and four gas gangrenes [12,14,15,17,18,19,20,21,22,23,24,25]. In the case of bacteremia, most cases have occurred in immunocompromised patients (17 cases) [14,18,19]. Bacterial mortality is most likely related to the production of a hemorrhagic toxin and proteinases. The *C. sporogenes* hemorrhagic toxin is maximally produced at the early growth phase, is produced well in a peptone- or ammonia-rich medium, and has a molecular weight different from that of other intestinal hemorrhage-inducing bacterial toxins released by *C. difficile*, *C. perfringens*, and *C. sordellii* [26]. Hara-Kudo et al. studies have shown that the hemorrhagic toxin has collagenase activity and is responsible for the hydrolysis of type III and collagen IV, the main component of blood vessels’ intima and tunica media. Therefore, the hemorrhagic toxin produced by *C. sporogenes* is an important virulence factor, and the hemorrhage induced by this toxin is related to its collagenase activity [26]. On the other hand, identifying *C. sporogenes* as a cause of bacteremia in an immunocompetent patient is very rare. In immunocompetent patients, a *C. sporogenes* infection has resulted from a secondary infection [12,15]. 

*Clostridium sporogenes* and *C. botulinum* Group I are closely related mesophilic bacteria with genotypic and physiological characteristics, including proteolytic properties and the ability to form spores of high thermal resistance. *Clostridium botulinum* Group I and *C. sporogenes* are responsible for foodborne, infant, and wound botulism [27]. Similar to *C. botulinum* group I and II strains, some *C. sporogenes* strains could produce botulinum neurotoxin serotype B. The genes encoding the production of all BoNT/B subtypes are organized in a haemagglutinin gene cluster. However, depending on the subtype present, it may be located within the chromosome or carried by mobile extrachromosomal elements [28]. Research by previous authors has shown that *C. sporogenes* strains carrying the *bont*/B1-B6 genes occurred in different geographical regions, e.g., France, Australia, the United Kingdom, Italy, and the United States [28,29,30,31,32,33]. Moreover, strains responsible for infant infections (strain no. CDC 1632 and AMA1195) [28,33] and infection of wounds (stain no. B2 450) have also been mentioned [32]. Brunt et al. have reported that the plasmid-borne subtypes BoNT/B1, B2, and B6 could be produced by *C. botulinum* and *C. sporogenes*. A comparative genomic study with 556 highly diverse strains of *C. botulinum* Group I and *C. sporogenes* (including 417 newly sequenced strains) has reported a core genome single-nucleotide polymorphism (SNP) analysis that revealed two significant lineages: *C. botulinum* Group I (most strains possessed botulinum neurotoxin gene(s) of types A, B, and F) and *C. sporogenes* (some strains possessed a type B botulinum neurotoxin gene). Of the 104 *C. sporogenes* strains identified, 20 isolates possessed a gene encoded botulinum neurotoxin or either subtype B1, B2, or B6. All subtype B1, B2, and B6 *bont* genes were in a *ha* neurotoxin gene cluster. Moreover, a new cluster containing the gene encoding the botulinum toxin subtype B1 has been identified in five *C. sporogenes* strains [27].

## 3. Double Nature of *Clostridium butyricum*

*Clostridium butyricum* is an obligate, anaerobic, rod-shaped Gram-positive bacterium, spore-forming in various environments, such as the human gastrointestinal tract and soil. These organisms have many applications in fuel industries as by-product producers and in medicine as probiotic strains, especially in Asia [34]. Butyric acid is the main chemical these bacteria produce in fermentation via the but–buk pathway of dietary fiber and other substances not digested by the human digestive system. Many studies indicate that short-chain fatty acids (SCAFs) produced in the intestines by the microbiota, which include butyric acid, influence the regulation of the homeostasis of the immune system and the functioning of the intestinal barrier [35]. Butyric acid is widely researched and known to have anti-inflammatory and anti-cancer properties. Table 1 showed the role of *Clostridium butyricum* in human health and disease. However, some strains of these organisms have also proved to be neurotoxigenic pathogens [8].

### 3.1. Clostridium butyricum as a Probiotic

*Clostridium butyricum* is commonly recommended as a probiotic after antibiotic therapy or surgical procedures. It was first isolated from pig intestines by Prażmowski in 1880 [36], and in 1933 it was isolated from the feces of healthy individuals by Dr Miyairi. Then, in 1963, *C. butyricum* MIYAIRI 588 (CBM 588) was isolated from the soil and found application as a probiotic strain. It was used in Japan as a drug to relieve gastrointestinal symptoms after antibiotic therapy, such as diarrhea. In 2014, the European Parliament authorized placing *Clostridium butyricum* (CBM 588) on the market as a new food ingredient under Regulation (EC) No 258/97 [37].

CBM 588 is entirely safe, which has been confirmed in laboratory tests on animals as a feed additive and in humans [38]. CBM 588 is resistant to stress effects such as low pH and antimicrobial agents [39]. After oral administration, the composition of the normal gastrointestinal microflora is regulated by increasing the beneficial microflora and reducing harmful strains of microorganisms. At the same time, it improves digestion and the functioning of the digestive system. They create an unfavorable environment for pathogenic organisms mainly by producing butyric acid and adhering to human epithelial cells, creating a protective mucosal barrier. It reduces the risk of infection development [39,40]. They can strengthen the intestinal immune response by stimulating the development of beneficial intestinal microflora [40]. A combination of probiotic strains can also be used to improve the effect of *Clostridium* as a probiotic. So et al. [41] investigated selecting specific strains of lactic acid bacteria (LAB) that could synergistically enhance the probiotic functions of *C. butyricum*. Supernatants of 249 lactic acid bacteria were examined, and observations were made that 24 strains did not inhibit the growth of *C. butyricum*. Additionally, 4 of these 24 strains induced a more than twofold promotion in the growth rate of *C. butyricum* during co-culture with this bacterial strain. This growth promotion was verified by qPCR. In particular, Lactobacillus brevis JL16 and Lactobacillus parabuchneri MH44 stimulated *C. butyricum* more effectively than other strains did [41].

CBM 588 is a probiotic bacterium that has already been used to prevent post-antibiotic diarrhea and in animal supplementation. Much research is still being conducted to elucidate the exact mechanism of protection of the intestinal epithelium by bacteria. CMB 588 is known to increase the abundance of Bifidobacterium, Lactobacillus, and Lactococcus and enhance intestinal barrier function in mice with dysbiosis after antibiotic therapy [40]. CMB 588 also significantly controls antibiotic-induced intestinal inflammation by increasing anti-inflammatory lipid metabolites such as palmitic acid, 15d-prostaglandin J2, and protectin D1 [40]. Studies also indicate that the administration of *Clostridium butyricum* effectively restores the intestinal microbial balance after colonoscopy and contributes to faster recovery [42].

*Clostridium butyricum* (CB) is also used as a supplement regulating the composition of the intestinal microflora and the quality of animal meat [43]. Zhang et al. investigated the effects of CB supplementation and rumen-protected fat (RPF) in increasing diet density and providing essential fatty acids on goat meat’s growth performance, nutritional value, and oxidative stability. The test results indicate that only in the case of shear force is there an observed interaction between CB and RPF. The content of intramuscular fat (IMF) is higher in the case of CB and RPF diets. Moreover, pH after 24 h and a* (redness) values, total antioxidant capacity, glutathione peroxidase activity, 18:3, 20:5, and total polyunsaturated fatty acid concentrations are increased, while L* (lightness) values, shear force, and malondialdehyde content (*p* = 0.044) are decreased by the addition of CB. In addition, CB supplementation increases the content of essential amino acids, flavor amino acids, and total amino acids. It also increases the expression of lipoprotein lipase and peroxisome proliferator-activated γ receptor (PPARγ) and decreases the expression of stearoyl-CoA desaturase (SCD). It should, therefore, be emphasized that the supplementation of CB and RPF in goats improves carcass characteristics, meat quality, and fat deposition in connection to increasing the expression of lipogenic genes in LT muscle [43]. Therefore, it is worth including these bacteria in the goat diet.

*C. butyricum* supplementation may also improve lipid metabolism and growth performance in piglets with intrauterine growth restriction (IUGR) and their suckling efficiency [44]. Zhang et al. investigated the effect of *Clostridium* supplementation on hepatic lipid disorders in IUGR-suckling piglets. The control sample consisted of piglets that received physiological saline added to milk. In contrast, the test sample consisted of piglets fed with milk with the addition of C. butyricum at a dose of 2 × 10^8^ colony-forming units (CFU)/kg body weight. The research results indicate that C. butyricum supplementation influences the intestinal microflora of IUGR piglets, reducing opportunistic pathogens in the ileum, such as Streptococcus and Enterococcus. The microorganisms hydrolyze bile salts, increasing bile acids. These can be transported to the liver and act as signaling molecules to activate the hepatic X receptor α (LXRα) and farnesoid X receptor (FXR). Therefore, reducing the number of these microorganisms accelerates the synthesis and oxidation of fatty acids. It lowers cholesterol levels, which improves the morphological condition of the liver in IUGR piglets, normalizes lipid metabolism, and improves the suckling efficiency of IUGR piglets [44].

Studies also indicate the hepatoprotective effect of CB in sea bass by reducing the activity of hepatic aspartate aminotransferase (AST) and increasing alkaline reaction phosphatase (AKP) and acid phosphatase (ACP) activity. Additionally, CB has a significant impact on strengthening the livers’ immunity. CB regulates the content of metabolic biomarkers such as arachidonate, crotonyl-CoA, and D-glucose 1-phosphate, which affects the main gluconeogenic, lipogenic, and amino acid metabolic pathways [45]. Research results indicate improved immunity and metabolism in sea bass, suggesting a hepatoprotective effect in humans [45].

### 3.2. Role of Clostridium butyricum in Health Promotion

More and more research indicates that the intestinal microflora influences metabolic syndrome development. One of the pathologies of this disease is obesity. Research suggests a negative correlation between the content of *C. butyricum* and the predisposition to the development of obesity, but the mechanism of this effect is still unclear. Five isolates of *C. butyricum* were administered (FYNDL1T1 (L1T1) and FHBSJZ1T1 (Z1T1) were isolated from the feces of cow and dog, respectively, strains FHLJZD47T7 (47T7) and NXYCHL3M3 (L3M3) were isolated from healthy volunteers, and strain C20_1_1 (C20) was isolated from an obese volunteer) to mice on a high-fat diet to determine their impact on the development of obesity. All tested isolates inhibited the formation and inflammation of subcutaneous fat, and two significantly reduced muscle mass gain and reduced dyslipidemia and liver steatosis [46]. Research indicates that a similar effect is not achieved by administering sodium butyrate, which suggests the action of microorganisms as factors inhibiting the development of obesity [46]. Research was undertaken to explain the effect of the CCFM1299 (C20_1_1) strain in preventing the development of obesity. For this purpose, CCFM1299 was orally administered to mice treated with a high-fat diet for 12 weeks. The results indicated that this strain inhibits the development of obesity by increasing energy expenditure. In addition, it increases the expression of genes related to the thermogenesis of brown adipose tissue (BAT). The term strain may also influence the expression of immune-related genes in epididymal white adipose tissue (eWAT). The immunomodulatory effect may be achieved by affecting the complement system, since factor D (CFD) gene expression was significantly decreased. The research results also indicate that the *C. butyricum* strain affects the metabolism of bile acids because increased concentrations of ursodeoxycholic acid (UDCA) in feces and taurohyodeoxycholic acid (THDCA) have been recorded in serum [47]. The presented results indicate the potential effective use of *C. butyricum* supplements in inhibiting the development of obesity [47]. 

One of the main complications of obesity is the development of diabetes. It is a chronic metabolic disease that can develop for a very long time and initially causes no apparent symptoms. It is associated with an increased glucose level in the blood due to the lack of insulin secretion, its reduced amount or tissue resistance to the action of insulin. If undiagnosed, untreated or treated incorrectly, it can cause damage to organs (eyes, heart, kidneys) and nerves.

Tayyib et al. examined the effects of *Clostridium butyricum* and magnesium supplementation on intestinal dysbiosis and blood sugar levels. The research was conducted on diabetic rats on an elemental diet. The control group received metformin, test group G1 received *Clostridium butyricum* (1.5 × 10^5^ CFU/day), test group G2 received magnesium (500 mg/kg/day), and the study group (G3) received *Clostridium butyricum* (1.5 × 10^5^ CFU/day) and magnesium (300 mg/kg/day). Blood glucose and magnesium levels and a complete blood count were tested. Additionally, blood glucose levels were randomly monitored twice a week for three weeks. The results indicate that *Clostridium butyricum* effectively balanced blood glucose levels compared to those of other groups. Moreover, it restores the dysbiosis of microorganisms [48]. Therefore, it is justified to introduce *C. butyricum* into the diet of people with type 2 diabetes [48].

It is known that intestinal dysbiosis influences the development of diabetes and its complications, but the mechanism of these is only partially known. Zhou et al. studied the relationship between the gut microbiota and vascular inflammation in diabetic mice [49]. The research results indicate that the amount of CB in diabetic mice was significantly lower compared to that in the control group. Additionally, impaired vascular function, inflammation in arterial tissue, and increased retained oxygen species were demonstrated. It is worth noting that CB administration rebalanced the intestinal microflora and protected vascular function in diabetic mice by activating the Nrf2/HO-1 pathway. Therefore, administering CB to people with diabetes may alleviate vascular changes and improve the intestinal microbiome.

Research indicates the wide use of CBM 588 in improving our health after various surgical procedures. There are many treatment methods in which the intestinal microflora plays a significant role. For example, in patients undergoing hematopoietic stem cell transplantation (HSCT), the intestinal microbiota plays a vital role in further prognosis, transplant effectiveness and complications, and the occurrence of graft-versus-host disease (GVHD). It has been shown that the administration *Clostridium butyricum* MIYAIRI 588 (CBM588) as a live biotherapeutic agent is associated with maintaining a normal intestinal microflora in the early period after HSCT. However, alpha species diversity decreased significantly in patients not treated with CBM588, while β diversity shows that CBM588 did not change intestinal microflora structure 7–21 days after HSCT. It is worth emphasizing that patients who developed GVHD showed structural changes in the microbiota compared to before the transplant, which was recorded 14 days before the development of GVHD. Enterococcus numbers significantly predominated in GVHD patients after HSCT, and the Bacteroides population did not change. However, in patients who received CMB588, Enterococcus and Bacteroides were reduced [50]. The results of the presented studies suggest that preoperative administration of CBM588 effectively maintains the balance of the intestinal microflora and improves the effectiveness of treatment [50].

Immune checkpoint inhibitors (ICIs) are the standard treatment for patients with advanced non-small cell lung cancer (NSCLC). This modern therapy benefits patients in increased progression-free survival (PFS) and overall survival (OS). Nevertheless, some patients experience primary or secondary resistance to ICI treatment, the reasons for which are not entirely clear [51,52,53]. These are related to the tumor’s molecular characteristics, the epigenetic profile, or the gut microbiome [54,55,56].

Clinical preliminary studies have shown a positive correlation between *Clostridium butyricum* MIYAIRI 588 (CBM588) supplementation and the effectiveness of ICIs in NSCLC treatment. Paz Del Socorro et al. tested in mouse models whether the strain could enhance the immunogenicity of tumor-draining lymph nodes to overcome ICI resistance [57]. They showed that CMB 588 improves the effectiveness of ICI (anti-programmed cell death protein 1, aPD-1) [57]. It is dependent on the acquisition of a regulatory phenotype of intestinal phagocytes that limits intestinal damage and accumulation of immunosuppressive Ror+ T_reg_ (Retinoic Orphan Receptor T_reg_) in tumor-infiltrating lymph nodes after PD-1 blockade [57]. The authors indicate that live CBM588 can suppress a subset of Rorγt+ T_regs_ in the colonic mucosa. Therefore, the enhanced response to PD-1 blockade in patients supplemented with the live biotherapeutic CBM588 may be supported by the more significant accumulation of immunosuppressive Rorγt+ T_reg_ to the colon, which may contribute to a more immunogenic reprogramming of tumor-draining lymph nodes that are strategically placed to infiltrate the tumor. In addition, the lowered frequency of Rorγt+ T_reg_ at the tumor-infiltrating lymph nodes is linked to microbiota-modulated tryptophan catabolism upon CBM588 treatment. Further, the CBM588-induced immunogenic conversion of the tumor-infiltrating lymph nodes is related to the Indoleamine 2,3-Dioxygenase 1/Inerleukine 10 (IDO1/IL-10) axis upon PD-1 blockade (IDO1 catalyzes the initial step in the degradation of tryptophan) [57].

What is more, it was observed by the authors that there is dysbiosis induced by anti-PD-1, and the beneficial impact of CBM588 on the effectiveness of PD-1 blockade is linked to lowered alpha diversity of the gut microbiota [57]. 

Saitsu et al. [58] also describe a case supporting polypectomy treatment with C. butyricum. It is known that polypectomy during pregnancy increases the risk of premature birth and even miscarriage. The study authors administered probiotics orally, including *Clostridium butyricum* and 17-alpha-hydroxyprogesterone caproate, to a 30-year-old pregnant woman in whom an approximately 40 mm cervical polyp was detected. As a result, the development of the polyp, which disappeared in the 28th week of pregnancy, was reversed. The patient gave birth to a healthy baby at term. Therefore, it can be concluded that probiotics can effectively prolong pregnancy in the case of pathology.

**Table 1:** The role of *Clostridium butyricum* in human health and disease.

*Clostridium botulinum* as a Health Support	Source
With oral administration, the composition of the normal gastrointestinal microflora is regulated by increasing the beneficial microflora and reducing harmful strains of microorganisms; improves digestion and the functioning of the digestive system.	[39]
Creates an unfavourable environment for pathogenic organisms mainly by producing butyric acid and adhering to human epithelial cells, creating a protective mucosal barrier.	[39]
Prevents post-antibiotic diarrhea.	[39]
LAB could synergistically enhance the probiotic functions of *C. butyricum*.	[40]
Administration of *C. butyricum* effectively restores the intestinal microbial balance after colonoscopy and contributes to faster recovery.	[41]
Negative correlation between *C. butyricum* content and the predisposition to the development of obesity, and potential effective use as supplements in inhibiting the development of obesity.	[45,46]
Effectively balances blood glucose, alleviates vascular changes, and improves the intestinal microbiome, suggesting support of type 2 diabetes treatment.	[47,48]
Improves the effectiveness of HSCT and maintains the balance of the intestinal microflora.	[49]
In non-small cell lung cancer patients, supports the effectiveness of treatment that uses inhibitors of immunological control points.	[56]
Supports polypectomy treatment.	[57]

### 3.3. Pathogenicity and Threats of Clostridium butyricum

While non-toxic strains of *Clostridium butyricum* are commonly used as probiotics and supplements to help treat many conditions, other strains can cause pathological conditions such as botulism in infants or necrotizing enterocolitis in premature infants. Some strains may also cause harmful effects on the intestinal mucosa. Moreover, the toxin gene has been identified based on genome sequencing [59].

It is also known that pets can also be a source of infection, such as a turtle, which was the source of infection in two infants in Ireland who were diagnosed with botulism [8].

Another threat that some strains of *C. butyricum* may cause is necrotizing enterocolitis (NEC). Clinical symptoms of varying degrees of severity include bleeding from the gastrointestinal tract, ulceration, and, consequently, necrosis of the mucous membrane, abdominal distention, gas in the portal venous gas, and pneumatosis intestinalis [59]. The mechanism of NEC formation is still unclear despite many studies on the pathogenesis of NEC. It is known that the bacterium most frequently involved in the pathogenesis of NEC belongs to the *Clostridium*, and the first association between *C. butyricum* and NEC was described in 1977. Unfortunately, the occurrence of NEC is associated with high mortality (20–30%); what is more, survivors experience long-term complications. The diagnosis and prevention of NEC is still difficult because the clinical symptoms and markers of NEC are not specific, which makes it difficult to diagnose the disease correctly. NEC is a multifactorial disease, and the susceptibility of premature infants to the disease is still unclear. The occurrence of NEC is caused by factors such as immaturity of the premature infant, enteral nutrition, and intestinal dysbiosis, which leads to an imbalance between pro- and anti-inflammatory factors [60]. Studies indicate an association between NEC and intestinal colonization by *C. butyricum*, *C. neonatale*, or *C. perfringens* in premature infants [60].

**Figure 2:**
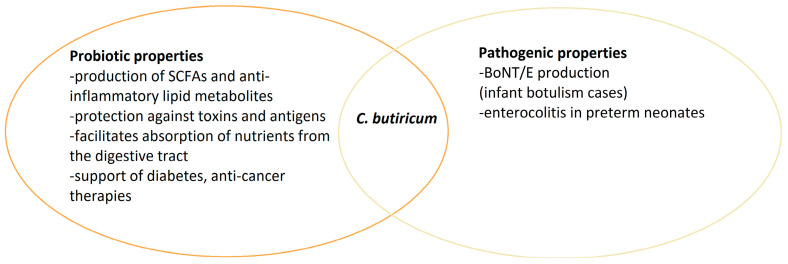
Dual nature of *C. butyricum* species.

*C. butyricum* has various properties and may have different health effects depending on the strain. Many studies indicate the probiotic and health-promoting effects of *C. butyricum* strains. Unfortunately, as in the case of other bacteria, some strains can have very unfavorable effects, even leading to death, especially in newborns and small children (Figure 2). Nevertheless, *Clostridium* is a part of the intestinal microflora, and understanding the relationships between other microorganisms and their impact on health is still a challenge for scientists.

## 4. Genetic Mechanism of *bont* Genes Expression in Non-*C. botulinum* Strains

The botulinum toxin gene has a rather complex molecular structure. There is a fixed element is the NTNH (non-toxic, non-haemagglutinin) fragment. It plays a protective role for the toxin NTNH, protecting the toxin from the acid environment in the stomach [61,62].

Smith et al. [63] studied the structure, distribution, and gene sequence of eight different toxin complexes representing four different BoNT/A subtypes (BoNT/A1-Ba4) and one BoNT/B1 responsible for most cases of botulism in humans [63]. The strains representing these subtypes were Hall Sanger ATCC 3502, CLB A1 ATCC 19397, CLC A1 Hall, and pCLD B1 okra. The gene arrangement within the three BoNT/A1 strains and the BoNT/B1 okra strain is identical in the orientation and composition of the individual functional and structural elements. It includes NTNH, BoNT, HA70, HA17, HA33, and BotR, (A1 or B1) [63].

Strains representing the BoNT/A2, BoNT/A3, and BoNT/A4 subtypes (CLM A2 Kyoto-F, pCLK A3 Loch Maree, pCLJ A4, respectively) contain the *orfX3*, *orfX2*, *orfX1*, *BotR*, *p47*, *ntht*, and *bont* genes. In the pCLJ bvB strain, BoNT/tvB is located in the plasmid, approximately 97 kb away from BoNT/A4 [63]. The different types of *bont* clusters have characteristic flanking sequences, which are not irrelevant for the horizontal transfer of *bont* genes. Thus, in strain A1, we find IS3 and flagellin sequences. In strain A2 sequences, *arsC*, and A3 IS3, IS605, and *lycA* are also present in strain A4. IS256 is present in bvB and B1, where functional flagellin sequences are also present.

*Clostridium botulinum* is divided into four groups (I–IV), where groups I and II cause human diseases, and III cause disease in animals. Human cases associated with Group III are extremely rare. An analysis of the genomes of groups I, II, and III has shown that the toxin genes, including the *bont* cluster, are carried by plasmids. With Group III, they are found within prophages. The Group III genomes contain many plasmids carrying various toxin genes. Some genes are also found in *Clostridium* species other than *C. botulinum*; some move between different plasmids within the same type [64].

*Clostridium botulinum* group I strains mainly have botulinum neurotoxin genes on their chromosome, while some genes (*bont/a*, *bont/b*, and *bont/f*) are located on plasmids. Sometimes, these genes are found within the chromosome, and in other strains, they are located within plasmids. It extends to *bont* genes of the same subtype in some cases. These *bont* gene clusters’ varied locations illustrate the different phases of horizontal gene transfer and demonstrate that *bont* gene location is a fluid.

Nawrocki et al. [65] carried out studies on the transfer of botulinum toxin genes between strains of the *Clostridium*. A pCLJ, a 270 kb plasmid encoding two BoNTs, was transferred from *C. botulinum* 657Ba (an auxotrophic donor strain created by multiple chemical mutagenesis). The study demonstrated the transfer of a 270 kb pCLJ plasmid containing two *bont* genes from the donor strain to various *Clostridium*. The frequency of transfer was highest to other *C. botulinum* group I strains. The plasmid was also transferred to non-toxic *Clostridium* species, namely *C. sporogenes* and *C. butyricum*. Ng et al. [66] have analyzed the genomic organization and evolutionary relatedness in four closely related A1 or A1(B) types of *C. botulinum* strains. They carried out an analysis of both the core botulinum toxin cluster and the surrounding functional genes. The authors indicate 90% similarity of core genes and 96% similarity of functional gene groups in these four genomes. Matching the genomes of the three A1 strains revealed a very similar chromosome structure with three small gaps in the ATCC 19397 genome and one additional gap in the Hall A genome, suggesting that ATCC 19379 is an evolutionary intermediate relationship between Hall A and ATCC 3502. Four gap regions indicated potential horizontal gene transfer and recombination events necessary for the evolution of A1 strains [66]. The authors’ analysis of the nearest region downstream of the HA+ cluster (B) in NCTC 2916 suggested possible recombination between HA+ (HA+ cluster for producing neurotoxin complexes composed with hemagglutinins (HA), a non-hemagglutinin non-toxic (NTNH), and BoNT proteins) clusters located on the plasmid and chromosome [66]. BoNT/A1 strains are unique in that the *bont*/A1 genes may be found as part of either orfX+ or ha+ gene clusters with this toxin subtype. The *bont*/A1 genes that are part of the ha+ gene cluster were inserted into an existing *bont*/(B) gene cluster via homologous recombination within the *bont*/B *ntnh* gene, producing a hybrid B-A *ntnh* gene and inserting the bont/A gene within the *bont*/B gene cluster [67]. In the case of NCTC 2916 and other BoNT/A1(B) strains, the silent (B) gene follows the *bont*/A1 gene cluster, but in HA+ BoNT/A1 strains, the *bont*/B genes are absent. No plasmids are present in these strains, so if the original gene transfer occurred between a plasmid-borne *bont*/A1 gene cluster and the chromosomally located *bont*/B gene cluster, the plasmid was subsequently lost. However, this is more likely between chromosomally located orfX+ bont/A1 gene clusters and ha+ *bont*/B gene clusters coexisting within the same chromosome. The potential sequence equality between serotypes in the IS3, IS256, IS605, *lycA*, *arsC*, and flagellin regions is interesting, which may influence the opportunity and strength of horizontal gene transfer [68]. It can be based on qPCR genotyping of *flaVR* variable regions [69]. Woudsta et al. [69] point to genetic variability in flagellin that may be geographically specific, and they made these conclusions by studying strains isolated from European and Canadian cases. In their study, the authors point out the genetic diversity of *flaVR* among *C. botulinum* strains and the clustering of *flaVR* types into five significant subgroups. Subgroups 1, 3, and 4 harbor proteolytic *Clostridium botulinum*, subgroup 2 consists exclusively of non-proteolytic *C. botulinum*, and subgroup 5 is specific to E-type *C. butyricum*. These are the conclusions of a study published in 2013. The BoNT-producing bacteria group currently includes numerous non-clostridial species (Table 2). The variable region of flagellin was useful in a study by Valdezate et al. [70] published in 2023. They studied the genetic diversity and phylogenetic relationships of *Clostridium botulinum* from foodborne botulism and infant cases. The botulinum toxin gene subtype (*bont*), the variable region of the flagellin gene (flaVR), and the seven-gene multilocus sequence type were examined by sequencing 37 bacterial cultures. It is well-known that botulism due to BoNT/B2 is prevalent in several Western European countries. The surprising finding is that some of the BoNT/B2 strains in France and Italy are *C. botulinum* group I, and some are *C. sporogenes* [70].

For each toxinotypes, the existence of *bont* genes in mobile genetic elements (except *bont*/C and *bont*/D) has been proven. Examples of subtypes BoNT/A2, BoNT/A3, BoNT/B1, BoNT/B2, BoNT/E1, BoNT/E3, and BoNT/E10 have also been described, in which the same botulinum clusters were demonstrated in the chromosome and in the plasmids. Extrachromosomal elements seem to be specific to certain metabolic groups of *C. botulinum* or other species of BoNT-producing Clostridia. For example, plasmids characteristic of *C. botulinum* group I (*C. parabotulinum*) are not significantly similar to plasmids of *C. botulinum* group II or *C. argentinense*, but also to sequences of *C. botulinum* group III (*Clostridium novyi sensu lato*) bacteriophages containing *bont*/C or *bont*/D. However, homology was observed between plasmids derived from *C. parabotulinum* and *C. sporogenes*. Smith et al. [71] conducted research using whole-genome sequencing (NGS), demonstrating that plasmids containing botulinum toxin genes can integrate into the bacterial chromosome, which may result in new strains of Clostridia stably producing BoNT [71]. The research was conducted using *Clostridium sporogenes* BoNT/B1 strain CDC 1632, *C. argentinense* BoNT/G strain CDC 2741, and *Clostridium parabotulinum* BoNT/B1 strain DFPST0006. Chromosomal *bont* gene clusters have been identified in the genomes of this bacteria in plasmid-like sequences or nested in large contigs, with no evidence of extrachromosomal elements [71]. The researchers demonstrate in the paper that full-length plasmid DNA carrying complete neurotoxin gene clusters has undergone integration into the chromosomes of three different bacterial species: *C. parabotulinum*, *C. sporogenes*, and *C. argentinense*. A fragment of the chromosomal sequence identified in *C. sporogenes* shared 99.5% identity with *bont*/B1-containing plasmid pNPD7 of *C. sporogenes* CDC 67071 [71]. 

Further, CDC 2741 contig AYSO01000020 contained a ~140 kb region, which shared 99.99% identity with plasmid pRSJ17_1 of *C. argentinense* BoNT/G strain 89G. At least DFPST0006 contig JACBDK0100002 contained a region that shared 100% identity with the *bont*/B1-containing plasmid pCLD of *C. parabotulinum* Okra. These studies show that not only the horizontal transfer of *bont* genes is vital for BoNT toxin production by strains other than *C. botulinum* but also the mechanisms that allow the integration of *bont* genes into their bacterial chromosome. It is an essential issue because integration into the chromosome can lead to a stable genetic construct, which strongly facilitates the identification by molecular biology methods of the presence of botulinum toxin-production genes. Plasmids carrying botulinum toxin genes can be temporarily lost; therefore, despite the occurrence of botulism’s clinical symptoms, identifying the cause of its onset is extremely difficult. On the other hand, the stable integration of *bont*-carrying plasmid fragments into the chromosome due to the biohazard of strains of the genus *Clostridium*, which are considered harmless and non-poisonous, seems to be a rather worrying phenomenon [71].

The other side of the BoNT story is that botulinum toxin is used in medicine, the pharmaceutical industry, and cosmetology. Despite being the most potent of the known biological toxins, it has applications in the treatment of spastic conditions, salivation, and neurological conditions, and is also used in oncological treatment [72]. Structural studies of BoNTs are critical. Safety in BoNT research is necessary for public health risk management, food preservation strategy development, and understanding toxinogenesis. 

## 5. Non-Clostridium BoNT-like Producing Strains

The *bont*-like gene cluster has also been observed in *Enterococcus* commensal bacteria in the gastrointestinal tract, both *Enterococcus faecium* and *Enterococcus faecalis* [9,73,74,75].

It has been proven that the gene cluster encoding BoNT/En (eBoNT/J) could be located on the conjugation plasmid of *Enterococcus faecium*, and BoNT/En cleaves both VAMP2 and SNAP-25 required for synaptic transmission in neurons, but differs from the sites of other known BoNTs [73]. BoNT/En does not appear to be toxic to mice. However, a chimeric toxin composed of the H chain of BoNT/A and the L chain of BoNT/En leads to paralysis. It induces symptoms of botulism, suggesting that this putative BoNT may have a different mechanism of action. The authors point out that the ability of common commensal strains of the Enterococcus genus to acquire genes that allow the production of botulinum toxin is highly dangerous, and the possibility of their emergence in multidrug-resistant strains appears to threaten biosafety, especially since *E. faecium* is responsible for multidrug-resistant hospital infections [73].

Additionally, Brunt et al. [9] identified the *bont*-like gene cluster in *Enterococcus.* They identified (by bioinformatics tools) and described a novel *bont* gene cluster from *Enterococcus* sp. 3G1_DIV0629, with a typical *ntnh* gene and an uncommon orfX arrangement. The sequence of this gene cluster shows that its closest relative is the *bont*/X cluster from the *C. botulinum* 111 strain. The amino acid sequence homology with BoNT/X is only nearly 39%. Still, modelling the 3D structure shows that the putative eBoNT/J is very similar to the neurotoxin BoNT/A structure. The authors indicate that further work is needed to investigate whether this structural variation will have important implications for the potential use of the putative eBoNT/J as a therapeutic agent [9].

Tehran et al. [74], in their review, point out that botulinum toxins are produced by bacteria other than *Clostridium botulinum*, which offers new opportunities for research in both the pharmaceutical and medical fields. Botulinum toxins produced by bacteria of the *Enterococcus* genus, as well as other non-clostridium bacteria that produce BoNT, may differ in structure and action [74].

In addition to BoNT-producing clostridia homologs, other taxa distinct from this genus have been identified as carrying the botulinum-like gene cluster. Such as *Chryseobacterium piperi* (*bont*/Cp1), *Enterococcus faecium*, or *Weissella oryzae*. However, the production of botulinum toxin by strains other than *Clostridium* has not been demonstrated. The discoveries mentioned above further complicate the taxonomic division of BoNT-producing bacteria [76].

Poulain et al. [75] note that the topic of the production of botulinum toxins—a very diverse group of toxins by bacteria, including those other than *Clostridium botulinum*—is still open. The latest molecular biology technologies have dramatically accelerated the work on understanding the action of BoNT and the spread of its genes between microorganisms. However, the subject is still topical, inexhaustible, and requires much research [75].

## 6. Problem with Classifications and Taxonomy

Different metabolic and physiological features cause the designation of bacteria able to produce botulinum toxins or carrying botulinum genes cluster highly problematic. Including all microbiological strains carrying genes that determine the production of botulinum toxins into a common framework is impossible. This phenotypic and genetic diversity is why classifying microorganisms containing the botulinum genes cluster has undergone many changes. Additionally, the mentioned *bont* gene cluster could be present in strains not taxonomically defined as *Clostridium* spp. This problem with taxonomic division does not exist in the case of *C. tetani* strains (it is known that both toxins, botulinum and tetanus, have the same typical ancestral toxin production-determining gene). In this case, the ability to produce the tetanus neurotoxin is specific and limited only to the mentioned species. Historically, BoNT-producing clostridia are defined as *C. botulinum* species, which, based on many discoveries made using biochemical and genetic tools, is not a correct definition and cannot be applied to all mentioned strains of this type. One of the earliest observations and division of the BoNT–producing clostridia was based on metabolic properties. It was noticed that some strains are proteolytic and non-proteolytic and that these strains differ from each other in lecithinase and lipase activity observed on egg yolk agar. On this basis, a division of the four metabolic groups was established. This grouping system was implemented to recognize the groups’ metabolic properties but did not make legitimate taxonomic changes. Moreover, this system showed that four distinct taxons should be highlighted. Over time, strains from species considered saprophytic (*C. baratii*, *C. sporogenes*) and even probiotic (*C. butyricum*) that were capable of producing botulinum toxin were discovered [1,2,76,77].

Genetic-based classification methods have evolved from DNA:DNA hybridization for 16S rRNA sequence analysis through pulsed-field gel electrophoresis (PFGE), multilocus sequence typing (MLST), amplified fragment length polymorphism (AFLP), up to whole-genome sequence analysis enabling average nucleotide identity (ANI) and single-nucleotide polymorphism (SNP) comparisons. These techniques proved that the above-mentioned groups could be confirmed as distinct species based on metabolic features [76].

The taxonomic changes of *Clostridium* spp. are still in progress (http://www.bacterio.net, accessed on 12 July 2024) [78,79].

The reclassification of *Clostridium* strains capable of producing botulinum toxin is still under discussion [79,80].

A new recommendation for taxonomic division was proposed by Smith et al. [79]. They proposed Latin binomial names for all members of each metabolic group. According to the authors, the new taxonomic division should include the following species: Proteolytic group I of *C. botulinum* should be changed to *Clostridium parabotulinum*; the designation of non-proteolytic group II should be changed to *C. botulinum*; the proposal for group III assumes changing the name to “*C. novyi sensu lato*”, closely related to *C. novyi* [60]. BoNT-producing clostridia’s remaining names remain (*C. argentinense*, *C. baratii*, *C. butyricum*, and *C. sporogenes*) [3]. This reclassification is not associated with the production of botulinum toxins. BoNT-producing strains can be distinguished by type or subtype classification, e.g., *C. parabotulinum* BoNT A1 or “*C. baratii* BoNT F” [79,81].

*Bont*-like genes are found not only in members of *Clostridium* spp. but also in other non-*Clostridium* strains, which makes the taxonomic division even more confusing, and it is impossible to limit BoNT-producing Clostridia to the species *C. botulinum*. As suggested by Smith et al. [79], the division of *Clostridium* strains capable of producing botulinum toxin takes into account genetic, metabolic, and phenotypic diversity and the diversity of subtypes within individual groups, which enable differentiation according to the type of toxin produced. However, new genetic discoveries suggesting the possibility of botulinum-like cluster genes in bacteria other than Clostridia means that the division, according to Smith et al. [79], does not exhaust the classification possibilities. Thus, taxonomic divisions evolve continuously with discoveries. A major challenge seems to be to include microorganisms possessing bont or bont-like genes into the appropriate taxa, taking into account their phenotypic and genetic features. Moreover, the greatest challenge seems to be understanding the mechanisms conditioning the appearance of the mentioned genes in microorganisms considered saprophytic, which is associated with ensuring microbiological safety in the food chain.

## 7. Conclusions

Despite its historical definition, *C. botulinum* is not the only species capable of producing botulinum toxins. As time passes and molecular biology tools develop, reports indicate the possibility of producing toxins or the presence of a botulinum cluster in bacteria that were not classified as *C. botulinum*. These findings highlight the need for a new taxonomic classification that is adequate to the current state of knowledge. Despite the updates, the taxonomic division will continue to evolve, which also creates the need to thoroughly understand the mechanisms determining the interspecies transfer of the botulinum cluster, as well as the need to develop methods for detecting bacteria predisposed to the production of botulinum toxins.

## Figures and Tables

**Table 2:** Division based on physiological and genetic differences between *C. botulinum* and other *Clostridium* strains. The other strains of BoNT-like producing bacteria.

Group of *C. botulinum* Species				Other Genera of BoNT-like Producing Bacteria			
				Other BoNT-Producing Clostridia			Organisms Potentially Able to Produce BoNT-like Proteins (I/Wo/J/En/Cp1 Toxin)
I	II	III	IV				
*C. botulinum* A and proteolytic strains of *C. botulinum* B and F	*C. botulinum* E and glucidolytic strains of *C. botulinum* B and F	*C. botulinum* C and D	*C. botulinum* G, which was assigned to a new species *C. argentinense.*	*C. baratii* Type F	*C. butyricum* Type E	*C. sporogenes* Type *B*	*E. faecalis* *Weisella oryzae* *Enterococcus faecium* *Chryseobacterium piperi*

## Data Availability

No data were created or analyzed in this study. Data sharing is not applicable to this article.
